# SERINC5 counters retroviruses and non-retroviruses

**DOI:** 10.3389/fcimb.2024.1516806

**Published:** 2025-01-20

**Authors:** Jinghua Yu, Chunyu Liu, Xinglong Qu, Xinglin Gao, Yue Liu

**Affiliations:** ^1^ Department of Echocardiography, The First Hospital of Jilin University, Jilin University, Changchun, Jilin, China; ^2^ Infectious Diseases and Pathogen Biology Center, The First Hospital of Jilin University, Jilin University, Changchun, Jilin, China; ^3^ Institute of Virology and AIDS Research, The First Hospital of Jilin University, Jilin University, Changchun, Jilin, China; ^4^ Department of Acupuncture, The Affiliated Hospital of Changchun University of Chinese Medicine, Changchun, Jilin, China; ^5^ Respiratory Department of the First Hospital of Jilin University, Jilin University, Changchun, Jilin, China; ^6^ Department of Urology, Siping Central People’s Hospital, Siping, Jilin, China

**Keywords:** SERINC5, human immunodeficiency virus 1, retroviruses, nonretroviruses, virus-host interaction

## Abstract

SERINC5 (serine incorporator 5), a member of the serine incorporator family, has been identified as a retrovirus restriction factor that inhibits the fusion of virions with the plasma membrane, thus blocking the release of the viral core into target cells and subsequently attenuating viral infectivity. Several viruses, such as human immunodeficiency virus (HIV), murine leukemia virus (MLV), and equine infectious anemia virus (EIAV), have evolved mechanisms to antagonize the host protein SERINC5 through HIV Nef, MLV glycosylated Gag, and the EIAV S2 protein. These viral proteins degrade SERINC5 on the cell surface through the endolysosomal system. In addition to its direct antiviral ability, SERINC5 also modulates immunity to inhibit the replication of retroviruses and nonretroviruses. This review summarizes the interaction between SERINC5 and viral replication, providing a promising avenue for fighting viral diseases.

## 1 Introduction

Efficient viral replication requires optimal circumstances in host cells. Viruses hijack the cellular machinery to facilitate their replication while the host deploys defense mechanisms to counteract viral infections. The dynamic interplay between viruses and their hosts plays a critical role in viral replication and pathogenicity.

Host’s restriction factors effectively inhibit viral replication. Restriction factors as pathogen recognition receptors (PRRs) often stimulate the immune system to produce interferon (IFN), and IFN, in turn, increases the level of restriction factors, which creates a positive feedback loop that helps the host eliminate viral infections (Firrito et al., 2018). Serine incorporator (SERINC) proteins exert antiviral function against retroviruses, including human immunodeficiency virus (HIV) (Rosa et al., 2015; Usami et al., 2015; Schulte et al., 2018), equine infectious anemia virus (EIAV) (Chande et al., 2016), and murine leukemia virus (MLV) (Ahi et al., 2016; Li et al., 2019b). But SERINC5 proteins are recognized as nonclassical host restriction factors because SERINC proteins are not induced by IFN (Xu et al., 2022; Wang et al., 2023).

**Figure 1:**
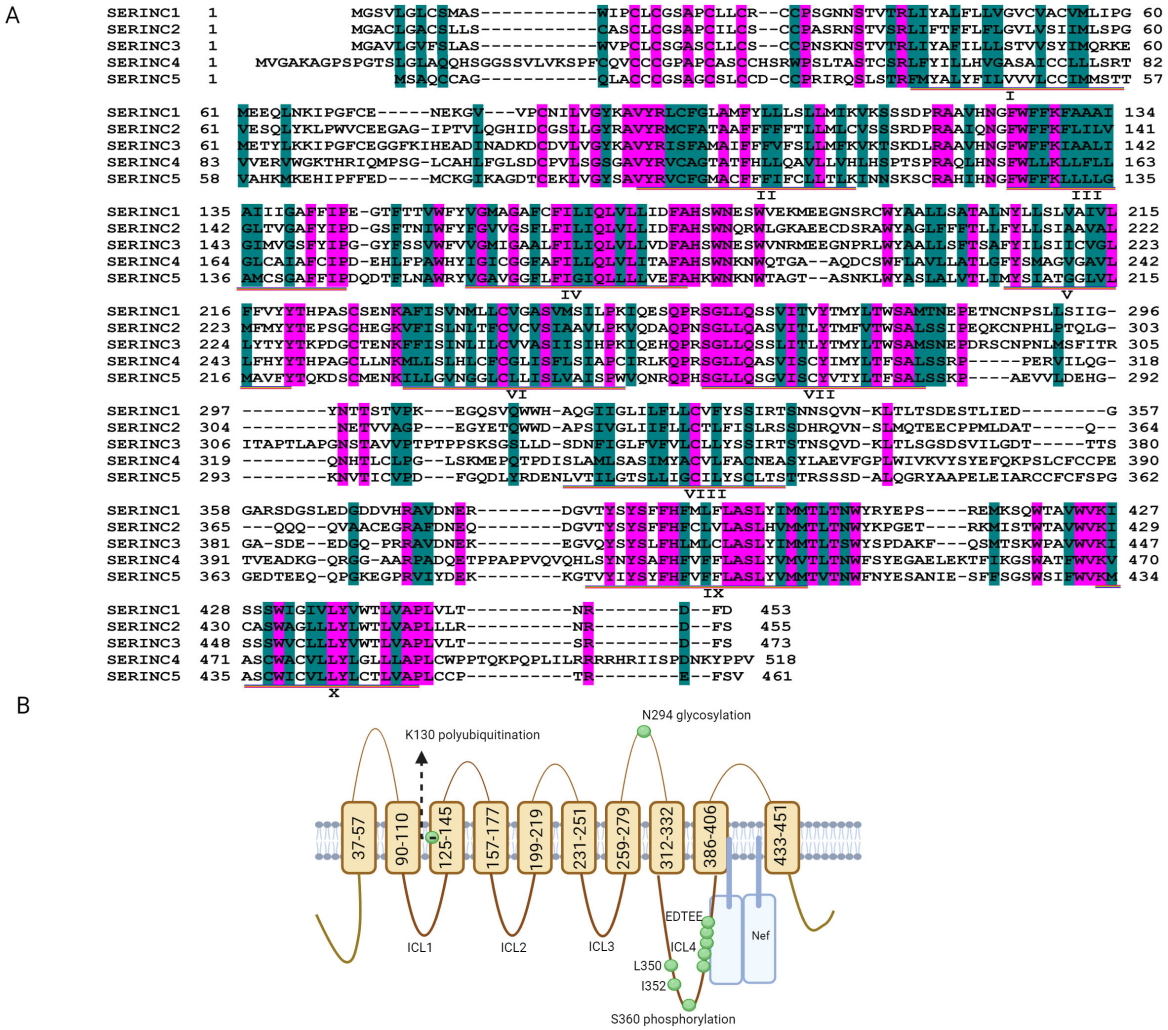
Putative topology of serine incorporator (SERINC) proteins. **(A)** The amino acid sequences of SERINC1, SERINC2, SERINC3, SERINC4 and SERINC5 were downloaded from https://www.ncbi.nlm.nih.gov/protein through searching NP_065806.1; NP_849196.2; NP_006802.1; NP_001244960.1; and NP_001167543.1, respectively. The sequences of SERINC1-5 were aligned with DNAssist version 2.2, and pink shading indicates that the amino acid sequences are identical. Green shading indicates that the amino acids have similar polarity, although the sequences are different, and the transmembrane domains of SERINC5 are labeled with I-X. **(B)** SERINC5 (isoform 001) is composed of 10 transmembrane domains, five extracellular loops, and four intracellular loops (ICLs) (Schulte et al., 2018) (https://www.ncbi.nlm.nih.gov/protein/NP_001167543.1). The E3 ubiquitin ligase Cullin3-KLHL20 targets SERINC5 for polyubiquitination at lysine 130 (K130). The stable expression of SERINC5 heavily relies on N294 glycosylation, and L350 and I352 in ICL4 are required to resist Nef. The phosphorylation at position 360 (S360) in SERINC5 induces a structural alteration in the ICL4 region that promotes the association of SERINC5 with Nef. The conserved EDTEE sequence in ICL4 maintains the ability of SERINC5 to resist Nef. **(B)** was generated via Biorender.

SERINC proteins are integral to the incorporation of serine into membrane lipids, facilitating the synthesis of phosphatidylserine and sphingolipids, crucial for cell membrane integrity (Inuzuka et al., 2005). As transmembrane proteins found across a diverse array of eukaryotic organisms, including animals, green plants, and fungi, five SERINC genes—SERINC1 to SERINC5—have been identified in humans (**Figure 1A**). Among these, SERINC5 has attracted considerable research interest due to its significant antiviral properties. SERINC5 has five alternatively spliced isoforms that share similar topologies but differ in the number of transmembrane domains and the length of the carbon terminal end (**Figure 1B**). SERINC5 is ubiquitously expressed across tissues, with a specific expression cluster in the liver (https://www.proteinatlas.org/ENSG00000164300-SERINC5/tissue), and endogenous SERINC5 mRNA are stable detected in human lung and intestine cells (Lai et al., 2022; Timilsina et al., 2022). The mRNA level of SERINC5 is expressed at significantly lower than GAPDH. Among SERINC5 isoforms, the longest isoform SERINC5-001 (461 aa, as shown in **Figure 1B**) mRNAs are higher (>10-fold) than other isoforms (Zhang et al., 2017), and its protein exhibits a half-life of approximately six hours (Zhang et al., 2017). Additionally, SERINC5 is upregulated during the differentiation of monocytes and in oligodendrocytes during myelination (Krueger et al., 1997; Zutz et al., 2020).

This review shows that SERINC5 restricts various viruses including HIV (Rosa et al., 2015; Usami et al., 2015), MLV (Ahi et al., 2016; Li et al., 2019b), EIAV (Chande et al., 2016), influenza A viruses (IAVs) (Lai et al., 2022; Zhao et al., 2022), SARS-CoV-2 (Timilsina et al., 2022), hepatitis B virus (HBV) (Liu et al., 2020), CSFV (Li et al., 2020) and Sendai virus (Zeng et al., 2021). Facing host’s restricting pressure, viruses have evolved themselves to overcome these restrictions through viral factors, such as Nef of HIV-1 (Rosa et al., 2015), S2 of EIAV (Chande et al., 2016), GlycoGag of MLV (Li et al., 2019b) and ORF7a of SARS-CoV-2 (Timilsina et al., 2022). In this review, the interaction between viruses and SERINC5 is described in depth for a range of viruses.

## 2 Retroviruses

### 2.1 HIV-1

HIV-1 is a virus that attacks the immune system, specifically CD4^+^ T cells, which help the body fight infections. Recently, broadly neutralizing antibodies have been explored as treatment and cure of HIV in clinical trials (Gruell and Schommers, 2022). According to neutralization sensitivity, HIV-1 isolates are categorized into three tiers (Seaman et al., 2010). Virus strains that are sensitive to neutralizing antibodies belong to tier 1, such as NL, SF162, HXB2, and 89.2, whereas virus strains that are resistant to neutralizing antibodies belong to tier 2 and titer 3, such as AD8 and JRFL (Zhang et al., 2019). Interestingly, tier 1 viruses are sensitive to SERINC5, whereas the majority of tier 2 and titer 3 viruses are resistant to SERINC5 (Zhang et al., 2019).

HIV-1 is a retrovirus from the *Retroviridae* family and *Lentivirus* genus that has an enveloped structure with an RNA genome. The viral genome encodes three structural proteins (Gag, Pol, and Env) and six accessory proteins (Tat, Nef, Vif, Rev, Vpr, and Vpu). In host cells, Env (gp160) is cleaved into gp120 and gp41 by the host protease. Upon infection of specific cells, HIV-1 utilizes the viral surface protein gp120 to bind with the host cell surface receptor CD4, and then, gp120 undergoes conformational alteration to expose gp41, which facilitates fusion between virions and cell membranes, allowing the release of the viral core particle into the cytoplasm for genome replication (Chen, 2019; Christensen et al., 2020).

#### 2.1.1 SERINC5 inhibits viral infection

**Figure 2:**
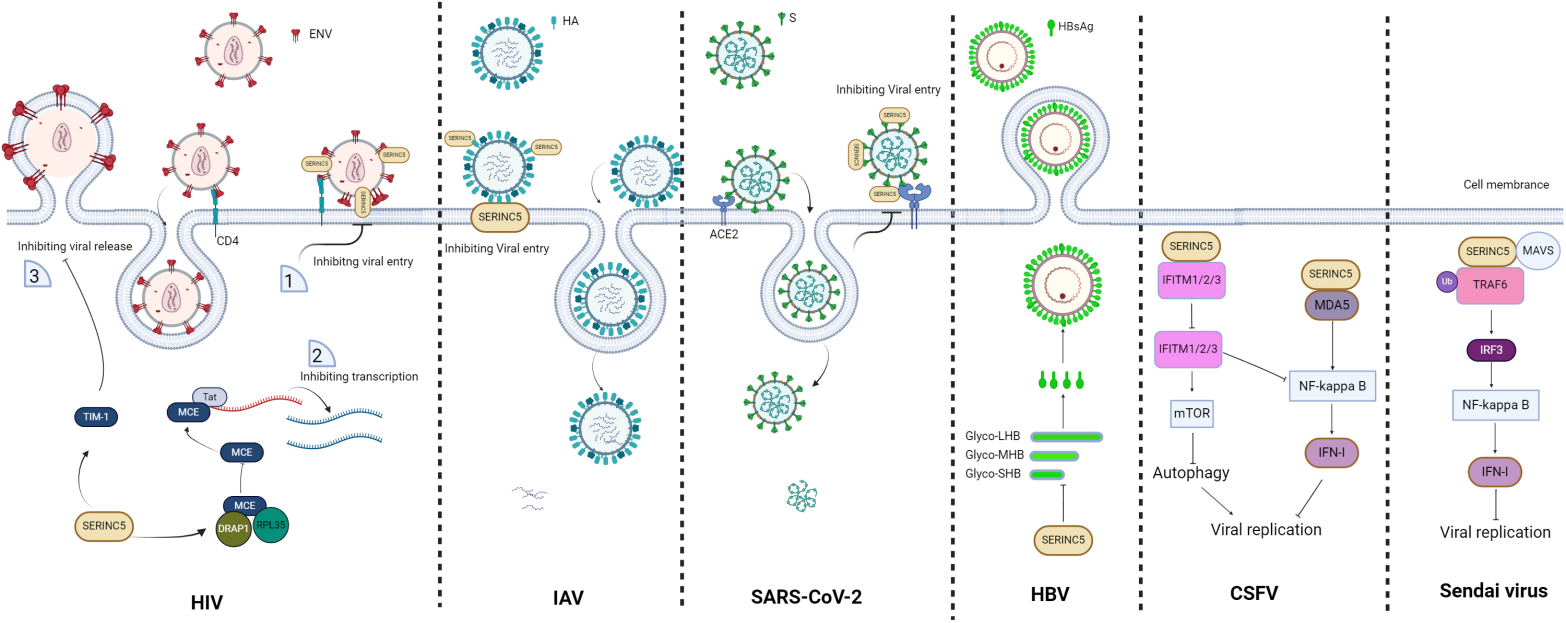
Schematic of the restriction role of SERINC5. For human immunodeficiency virus (HIV), virions without SERINC5 can easily infect host cells, whereas virions with SERINC5 cannot; moreover, SERINC5 inhibits viral transcription and virion release. For influenza A viruses (IAVs), SERINC5 in the cell membrane disrupts the infectivity of IAV by affecting membrane fusion and virus entry. For severe acute respiratory syndrome coronavirus 2 (SARS-CoV-2), SERINC5 in the virion inhibits viral entry. For hepatitis B virus (HBV), the presence of SERINC5 results in an increase in the nonglycosylation status of the large (LHB), middle (MHB), and small (SHB) subunit proteins and causes a reduction in HBsAg, consequently leading to a decrease in HBV secretion. For classical swine fever virus (CSFV), SERINC5 inhibits CSFV by interacting with the RNA sensor protein MDA5 and enhancing the MDA5-dependent IFN-I response, whereas SERINC5 interacts with IFN-induced transmembrane proteins 1/2/3 (IFITM1/2/3) to inhibit IFITM1/2/3-dependent autophagy and NF-κB inhibition, further leading to virus inhibition. For the Sendai virus, SERINC5 restricts the Sendai virus by promoting the production of IFNs through interactions with tumor necrosis factor receptor-associated factor 6 (TRAF6). The figure was generated via Biorender. ERGIC, endoplasmic reticulum-Golgi intermediate compartment.

SERINC proteins were identified as carrier proteins responsible for the incorporation of serines into various membrane lipids, including phosphatidylserine and sphingolipids (Inuzuka et al., 2005); nevertheless, SERINC5 did not regulate the lipid composition within HIV-1 particles (Trautz et al., 2017). In fact, SERINC5 present in the plasma membrane is effectively integrated into emerging HIV-1 virions, leading to a disruption in subsequent fusion with target cells (Rosa et al., 2015; Usami et al., 2015) (**Figure 2**). SERINC5 hinders the fusion of HIV with cell membranes (Usami et al., 2015; Beitari et al., 2017; Ward et al., 2020) by inducing the functional inactivation of Env glycoproteins (Sood et al., 2017), interfering with Env protein clusters (Zhang et al., 2019; Chen et al., 2020), and changing the conformation of gp120 (Featherstone and Aiken, 2020) on HIV-1 particles. Moreover, SERINC5 disrupts membrane asymmetry, which is closely associated with alterations in the structure of Env and a decrease in viral infection ability (Leonhardt et al., 2023). However, some HIV strains are resistant to SERINC5 (Zhang et al., 2019), partly because Env of these HIV strains cannot be inactivated by SERINC5 (Rosa et al., 2015; Usami et al., 2015; Beitari et al., 2017; Zhang et al., 2019; Timilsina et al., 2020).

#### 2.1.2 SERINC5 inhibits viral replication

A recent study demonstrated SERINC5 has the role in decreasing HIV mRNA levels (Shi et al., 2023).The ability of SERINC5 decreasing mRNA level is limited to the certain mRNA that are from lentivirus and plasmid DNA. Whereas the mRNA that are from cellular genes or foreign RNAs is not be decreased by SERINC5, for example, SERINC5 does not decrease the genomic level of SARS-CoV-2, which is foreign RNA (Shi et al., 2023). Although SERINC5 decreasing the mRNA level of HIV is confirmed, the molecular mechanisms are not elaborated by this study (Shi et al., 2023).

Meanwhile Ramdas and Chande also found that in the myeloid lineage cells SERINC5 reduces the level of HIV mRNA located in the cytoplasm (Ramdas and Chande, 2023). Host mammalian capping enzyme (MCE) is responsible for capping mRNA, while upon HIV infection, MCE is hijacked by HIV-1 Tat protein to the HIV transcriptional complex for capping HIV mRNA during viral transcription (Chiu et al., 2001). Viruses containing SERINC5 increase the mRNA levels of ribosomal protein L35 (RPL35) and the transcriptional repressor DRAP1 to increase the expression of them (Ramdas and Chande, 2023). Then, RPL35 and DRAP1 bind with MCE to inhibit MCE function in capping HIV mRNA (Ramdas and Chande, 2023), thus hinder viral protein production and the formation of progeny virions (Ramdas and Chande, 2023). However, these findings do not reveal how SERINC5 in virions regulates the transcription of RPL35 and DRAP1. It is speculated that virions with SERINC5 can activate intracellular signal transduction for the transcription of RPL35 and DRAP1, that virions with SERINC5 can be endocytosed and then SERINC5 can promote their transcription, or that endocytosed SERINC5 can activate endogenous SERINC5 to regulate the transcription of RPL35 and DRAP1 (**Figure 2**).

#### 2.1.3 SERINC5 inhibits viral release

T-cell immunoglobulin and mucin domain-containing proteins (TIMs), including TIM-1, TIM-3, and TIM-4, are type I transmembrane glycoproteins (Su et al., 2008). As a host restriction factor, the TIM-1 protein inhibits the release of HIV-1 and other enveloped viruses (Li et al., 2014). SERINC5 stabilizes TIM-1 expression by extending its half-life, further to strengthen the function of TIM-1 in inhibiting HIV-1 release (Li et al., 2019a) (**Figure 2**). Nef proteins of HIV-1 and other lentivirus proteins, such as MLV glycoGag and EIAV S2, have the function as antagonists to overcome TIM-1-mediated restriction (Li et al., 2014), and it is speculated that HIV Nef, MLV glycoGag and EIAV S2 resist TIM-1 possibly through degrading SERINC5.

#### 2.1.4 SERINC5 regulates viral infection-induced immunity and inflammatory responses

SERINC5 has been shown to directly inhibit viral production through targeting the viral life cycle. A recent study highlighted the crucial involvement of SERINC5 in innate immune responses, demonstrating its ability to increase IFN-I production and NF-κB signaling (Zeng et al., 2021). SERINC5 interacts with the outer mitochondrial membrane protein MAVS (mitochondrial antiviral signaling) and the adaptor protein TRAF6 (tumor necrosis factor receptor-associated factor), and they cooperatively promote IFN-I production and NF-κB activation (Zeng et al., 2021). Moreover, in myeloid target cells, SERINC5 incorporated into virions promotes the innate immune recognition of HIV-1 particles and proinflammatory cytokine production (Pierini et al., 2021). The incorporation of SERINC5 into virions renders HIV-1 more sensitive to some broadly neutralizing antibodies because SERINC5 in virions increases the ability of the neutralizing antibody 4E10 to bind to the gp41 membrane-proximal region (Beitari et al., 2017). However, the mechanism by which SERINC5 increases susceptibility to neutralizing antibodies is still unclear. It is speculated by the Tedbury team that in the presence of SERINC5 but not Nef, SERINC5 might slow Env refolding and prolong the time needed to bind neutralizing antibodies, or SERINC5 may promote structural modifications of Env and facilitate the binding of neutralizing antibodies (Tedbury and Sarafianos, 2017). Therefore, SERINC5 is crucial for modulating immunity and the inflammatory response to fight HIV, in addition to directly inhibiting viral production.

#### 2.1.5 Viral factors antagonize SERINC5

**Figure 3:**
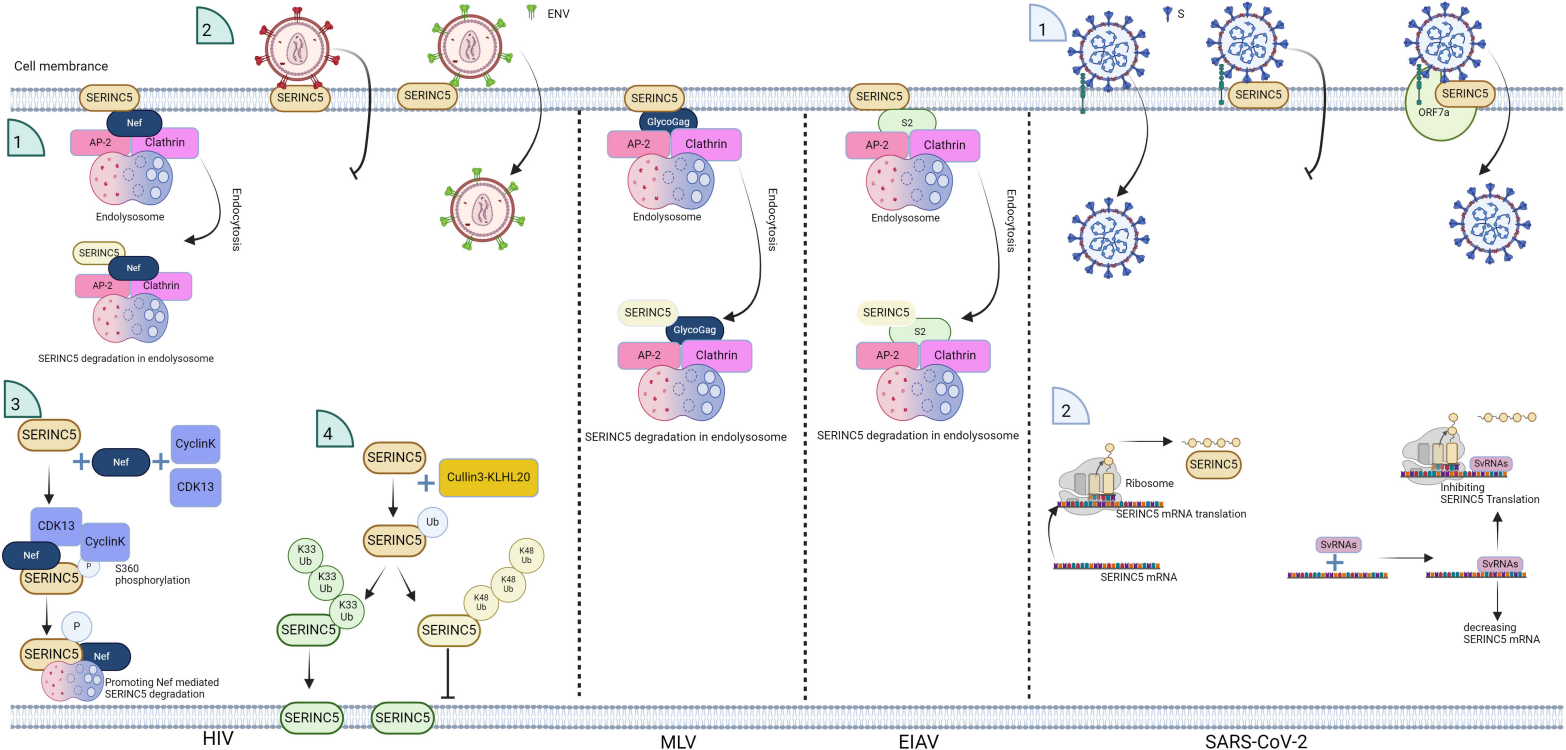
Schematic of the antagonistic effect of viruses on SERINC5. HIV Nef, murine leukemia virus (MLV) GlycoGag, and equine infectious anemia virus (EIAV) S2 promote SERINC5 degradation and inhibit SERINC5 incorporation into virions, and for HIV, the E3 ubiquitin ligase Cullin3-KLHL20 targets SERINC5 for polyubiquitination at lysine 130, which involves both K33- and K48-linked ubiquitin chains. K33-linked polyubiquitination promotes SERINC5 expression on the plasma membrane, and K48-linked polyubiquitination contributes to SERINC5 downregulation from the cell surface. CyclinK/CDK13 phosphorylates S360 in SERINC5, promoting the interaction between SERINC5 and Nef. IFN-I stimulation enhances the membrane surface levels of endogenous SERINC5. For SARS-CoV-2, the SARS-CoV-2 viral protein ORF7a binds with SERINC5 to inhibit SERINC5 function; svRNAs from SARS-CoV-2 interact with the 3’UTR of SERINC5 mRNA, leading to the suppression of SERINC5 expression in experimental settings. The figure was generated via Biorender.

Nef is a 27-kDa myristoylated HIV viral protein. Nef is a crucial factor in viral pathogenesis and disease progression (Pereira and daSilva, 2016). Still, Nef is not an essential factor for HIV replication because Nef-defective HIV virions have been isolated from long-term nonprogressive patients (Piguet et al., 1999), *in vitro* Nef-deficient virions have been constructed (Usami et al., 2015). However, Nef can downregulate CD4 (Piguet et al., 1999) and major histocompatibility complex (MHC) class I (Oldridge and Marsh, 1998; Blagoveshchenskaya et al., 2002), increase virion infectivity (Münch et al., 2007; Ramirez et al., 2023) and alter intracellular signal transduction pathways (Foster et al., 2011) to support HIV replication. When SERINC5 restricts HIV, Nef counteracts SERINC5 *in vitro* (Rosa et al., 2015; Usami et al., 2015; Cano-Ortiz et al., 2023). Nef possibly binds with ICL4 of SERINC5, which determines the sensitivity to Nef (Dai et al., 2018); however, whether ICL4 is the sole point of contact between Nef and SERINC5 needs further investigation. SERINC5 is distributed mainly to the plasma membrane and scarcely colocalizes with Rab5+ (early endosomes), Rab7+ (late endosomes), or Rab11+ (recycling endosomes) endosomes. However, in the presence of Nef, Nef interacts with SERINC5 and localizes SERINC5 to the Rab5+, Rab7+, and Rab11+ endosomes. These endosomes fuse with lysosomes for SERINC5 degradation (Shi et al., 2018). The degradation of SERINC5 further prevents SERINC5 from being incorporated into the budding virus (Aiken, 2015; Rosa et al., 2015; Usami et al., 2015; Heigele et al., 2016; Shi et al., 2018). In the absence of Nef, SERINC5 is packaged into virions, and SERINC5 in virions inhibits virion−membrane fusion after binding to the host receptor (Rosa et al., 2015; Usami et al., 2015) (**Figure 3**). Natural mutations or differences in Nef usually affect its antagonistic activity to SERINC5 (Jin et al., 2020; Toyoda et al., 2020; Kruize et al., 2021). Three conserved residues (Leu112, Tyr115, and Phe121) in Nef are required by the Nef homodimer, which is required for SERINC5 degradation (Staudt and Smithgall, 2020). The conserved sequences in Nef, such as dileucine motifs (ExxxLL) and carboxy-terminal diacid residues (EDAA), facilitate its binding with endocytic adaptor protein complexes (APs) 1 and 2 (Craig et al., 2000), and the binding of Nef with AP1/AP2 leads to continued progression toward the endolysosomal degradation of SERINC5.

Besides Nef, Env also resists the SERINC5 restriction. HIV NL strain is sensitive to SERINC5, whereas HIV AD8 strain is resistant to SERINC5 (Zhang et al., 2019). When the Env of NL (sensitive) and AD8 (resistant) is exchanged, the sensitivity to SERINC5 is changed (Beitari et al., 2017); therefore, the Env of some HIV-1 strains can overcome SERINC5 inhibition (Rosa et al., 2015; Usami et al., 2015; Beitari et al., 2017; Zhang et al., 2019; Timilsina et al., 2020). Moreover, SERINC5 does not inhibit pseudotyping with Env glycoproteins of the Ebola virus or vesicular stomatitis virus (Rosa et al., 2015; Diehl et al., 2021); therefore, other viral Envs can also resist SERINC5. Nef counteracts endogenous SERINC5 but not the much higher level of ectopic SERINC5, while HIV-1 Env is able to resist high levels of SERINC5. Neither Env nor Nef affects the incorporation of high levels of ectopic SERINC5 into HIV-1 particles, whereas HIV-1 Env, but not Nef, is able to resist high levels of SERINC5 in viral particles (Beitari et al., 2017). In Env there are five variable regions (V1–V5); V1, V2 and V3 are involved in trimer association; and the V1 and V2 loops are important for HIV Nef responsiveness and neutralization sensitivity (Usami and Göttlinger, 2013) and determine the ability of Env to counteract SERINC5 (Usami et al., 2015). Moreover, the V3 loop is also required for Nef activity in antagonizing SERINC5 (Beitari et al., 2017). Meanwhile, truncation of the Env cytoplasmic tail alters the conformation of Env and confers resistance to SERINC5 restriction (Haider et al., 2021; Xu et al., 2022). These results indicate that Env is a viral determinant of sensitivity to SERINC5.

In recent years, studies have shown that the viral core has an impact on sensitivity to SERINC5; for example, the Mason Pfizer monkey virus glycoproteins with MLV cores or the Mason Pfizer monkey virus cores are sensitive to SERINC5, but the cores of HIV are resistant to SERINC5 (Diehl et al., 2021).

Therefore, the virus successfully evades the host’s SERINC5 restriction by utilizing the different viral factors Nef, Env, and the viral core.

#### 2.1.6 Host factors modulate SERINC5 activity

The stability of SERINC5 is very important for SERINC5’s anti-HIV function, some domains and amino acids of SERINC5 determines its stability. The specific domain of SERINC5, known as ICL4, is crucial for maintaining the stability of SERINC5 when SERINC5 interacts with Nef, especially L350 and I352 in ICL4, which are required to resist Nef (Dai et al., 2018). The mutant SERINC5-F397L exhibits the lowest antiviral activity (Pye et al., 2020; Leonhardt et al., 2023). Removing the conserved EDTEE sequence from this loop decreases the stability of SERINC5 in the presence of Nef, but it does not affect the inhibitory activity or stability of SERINC5 in the absence of Nef (Stoneham et al., 2020). The stable expression of SERINC5 heavily relies on N294 glycosylation, as SERINC5 is highly susceptible to proteasomal degradation in the absence of N-glycosylation, but N294-glycosylation does not affect SERINC5 intrinsic restrictive activity or sensitivity to Nef (Sharma et al., 2018). The aromatic residue 412 in the fifth extracellular loop of SERINC5 plays a crucial role in improving the antiviral ability of SERINC5 against a range of retroviruses, including HIV-1, HIV-2, and simian immunodeficiency viruses (Tan et al., 2021).

In addition to viral factors regulating SERINC5, host factors also regulate the stability and function of SERINC5. In certain cellular environments, the expression or the function of SERINC5 is increased within host cells; for example, IFN-I stimulation increases the membrane surface levels of endogenous SERINC5 because intracellular SERINC5 is relocated to and stabilized on the plasma membrane (Passos et al., 2019). Moreover, upon the differentiation of cells toward the myeloid lineage, the level of SERINC5 is specifically upregulated (Zutz et al., 2020). Furthermore, the presence of CD4 enhances the Env-Ser5 interaction and helps SERINC5 dissociate Env trimers, further blocking viral entry (Zhang et al., 2019). Under certain circumstances, SERINC5 is decreased; for example, CyclinK/CDK13 phosphorylates a serine residue at position 360 (S360) in SERINC5, causing a structural change in the ICL4 region that enhances the interaction between SERINC5 and Nef. CDK13 interacts with SERINC5 only in the presence of Nef, which acts as an adaptor that connects SERINC5 with CyclinK//CDK13. This phosphorylation is essential for the Nef-mediated removal of SERINC5 from the cell membrane and the inhibition of SERINC5’s antiviral function (Chai et al., 2021). The E3 ubiquitin ligase Cullin3-KLHL20 targets SERINC5 for polyubiquitination at lysine 130 through a mechanism that involves both K33- and K48-linked ubiquitin chains, regardless of the presence or absence of a virus. K33-linked polyubiquitination promotes SERINC5 expression on the plasma membrane, whereas K48-linked polyubiquitination contributes to SERINC5 downregulation from the cell surface (Li et al., 2022a); therefore, through different types of polyubiquitination, SERINC5 expression on the plasma membrane is regulated positively or negatively.

### 2.2 MLV

MLV is a gamma-retrovirus (Rein, 2011). The amount of SERINC5 determines the degree to which the MLV is inhibited, and SERINC5 can lower the infectivity of the MLV in the absence of glycosylated Gag (glycoGag) (Ahi et al., 2016). GlycoGag is an accessory protein of the MLV that decreases the level of SERINC5 in a manner dependent on endosomal/lysosomal mechanisms (Usami et al., 2014). GlycoGag binds with SERINC5, which leads to the relocation of SERINC5 from the plasma membrane to the intracellular endosomal/lysosomal compartment (**Figure 3**). The amino acid sequence Y36XXL39 of glycoGag is required for SERINC5 translocation, and the P31 and R63 residues in glycoGag are essential for SERINC5 degradation (Li et al., 2019b). The potent antiviral ability of SERINC5 on the MLV has been confirmed in transgenic mice (Timilsina et al., 2020).

### 2.3 EIAV

EIAV, which only infects members of the Equidae (including horses, donkeys and mules), belongs to the *Lentivirus* genus of the *Retroviridae* family (Wang et al., 2023). SERINC5 inhibits EIAV replication, although with lower potency than SERINC5- inhibition of HIV-1 (Chande et al., 2016). However, the EIAV S2 protein, a dispensable accessory protein, relocates SERINC5 to the late endosomal compartment for SERINC5 degradation (Chande et al., 2016) (**Figure 3**). Similar to Nef, the myristoylation of S2 at the glycine 2 site is essential for its interaction with SERINC5, and myristoylation at the glycine 2 determines SERINC5’s plasma membrane localization (Chande et al., 2016; Ahmad et al., 2019). The leucine residue at position 26 of S2 is also necessary for the internalization of SERINC5 by S2, leading to a reduction in SERINC5 protein expression (Ahmad et al., 2019). Within a putative ExxxLL motif of S2, two leucine residues determine S2’s SERINC5-antagonising ability, mutation of two leucines leads to S2 losing the ability to promote viral infection in the presence of SERINC5 (Chande et al., 2016). Moreover, EIAV Env is also responsible for resistance to SERINC5 (Chande et al., 2016; Ahmad et al., 2019).

Therefore, it is concluded that the retroviruses HIV-1, EIAV, and MLV have similar abilities and mechanisms to antagonize the host factor SERINC5 (Ahmad et al., 2019).

## 3 Non-retroviruses

### 3.1 IAV

IAVs are enveloped viruses containing eight single-stranded, negative-sense RNA gene segments belonging to the *influenza virus A* genus within the *Orthomyxoviridae* family. IAV virions are surrounded by a lipid bilayer that consists of three viral transmembrane proteins (hemagglutinin (HA), neuraminidase, and matrix 2), which are essential for viral entry and assembly. HA is the most abundant surface protein in the virion and comprises two subunits, HA1 and HA2 (Weis et al., 1988; Gamblin et al., 2004), and HA proteins of the virion bind to the sialic acid receptor of the host cell, leading to viral uptake via endocytosis (Hu et al., 2020). With the disassembly of the IAV capsid, viral RNA is subsequently delivered into the cytoplasm (Zhao et al., 2022).

Initially, the infectivity of pseudo-virions typed with IAV glycoproteins and the HIV core is decreased by SERINC5 (Diehl et al., 2021). Then, two teams confirmed that SERINC5 directly restricts viral strain of IAV (Lai et al., 2022; Zhao et al., 2022). SERINC5 expressed in infected cells exerts anti-IAV function, and SERINC5 in infected cells does not affect the attachment of virions to the cell membrane surface but inhibits virion−cell fusion and viral RNA release by preventing IAV disassembly (Zhao et al., 2022). SERINC5 demonstrates a significant interaction with HA at the plasma membrane. Notably, the K130A mutation of SERINC5 results in a diminished localization of the protein to the plasma membrane. This altered localization is critical, as the presence of SERINC5 at the plasma membrane is essential for its effective interaction with HA (Lai et al., 2022). Moreover, various subtypes of influenza HA exhibit varying degrees of sensitivity to SERINC5 inhibition, whereas glycosylation sites in the HA protein of IAV are correlated with sensitivity to SERINC5. Mutation of specific HA glycosylation sites decreases the antiviral activity of SERINC5 against IAV (Zhao et al., 2022). SERINC5 is present in viral pellets (Lai et al., 2022), which means that SERINC5 might be incorporated into IAV virions, and whether SERINC5 in virions inhibits viral entry needs to be confirmed in the future. With respect to the regulatory effect of IAV on SERINC5, IAV does not encode an accessory protein to counteract the restriction of SERINC5 (Lai et al., 2022).

### 3.2 SARS-CoV-2

SARS-CoV-2 is the virus responsible for the ongoing coronavirus disease 2019 (COVID-19) pandemic. SARS-CoV-2 is an enveloped virus with a positive-sense, single-stranded RNA genome belonging to the *Betacoronavirus* genus within the *Coronaviridae* family. The genome of SARS-CoV-2 contains 14 functional open reading frames that encode various types of proteins, including nonstructural, accessory, and structural proteins. Structural proteins, including the spike protein (S), envelope protein (E), membrane protein (M), and nucleocapsid protein (N), are responsible for viral assembly and the formation of the viral shell (V’kovski et al., 2021). The S glycoprotein mediates the fusion of the virion-host cell membrane during the initial stages of viral particle entry (Jackson et al., 2022), and the S protein is assembled as a homotrimer in the virion surface. SERINC5 restricts S protein-mediated entry by blocking virus-cell fusion during SARS-CoV-2 infection (**Figure 2**), and the ability of SERINC5 to inhibit S protein-mediated entry is negated by the SARS-CoV-2 viral protein ORF7a (Timilsina et al., 2022), which is based on the complex formation of the SERINC5, S and ORF7a (**Figure 3**).

Both DNA and RNA viral genomes usually encode noncoding small viral RNAs (svRNAs), such as miRNAs that are 19–28 nucleotides (nt) long (Skalsky and Cullen, 2010), which can bind to the 3′ untranslated regions of targeted mRNAs, further regulating targeted mRNA expression. After SARS-CoV-2 infection, two svRNAs were investigated: svRNA 1 (24 nt) is located in the intergenic sequence between the N and ORF10 genes, whereas svRNA 2 (24 nt) is located in the N gene of the SARS-CoV-2 genome. Research has revealed that svRNA 1 and svRNA 2 interact with the 3’UTR of SERINC5 mRNA, leading to the suppression of SERINC5 expression in experimental settings (Meseguer et al., 2023) (**Figure 3**). Therefore, upon SARS-CoV-2 infection, SERINC5 inhibits virus-cell fusion, whereas the viral protein ORF7a and svRNAs antagonize SERINC5.

Recent studies present contrasting findings regarding the effect of SARS-CoV-2 infection on SERINC5 expression. Timilsina et al. (Timilsina et al., 2022) report that SARS-CoV-2 does not alter SERINC5 transcription levels at various post-infection time points in Calu-3 cells. In contrast, Meseguer et al. (Meseguer et al., 2023). observe a reduction in SERINC5 mRNA levels in COVID-19 patients, as well as a decrease in SERINC5 mRNA at 4 hours post-infection and a decline in SERINC5 protein levels at 16 hours post-infection in Vero E6 and HEK293T-hACE2 cells. These discrepancies underscore the need for further investigation into the regulation of SERINC5 expression by SARS-CoV-2, particularly considering the variability across different cell lines and antibodies.

### 3.3 HBV

HBV is a small enveloped DNA virus belonging to the *Orthohepadnavirus* genus of the *Hepadnaviridae* family. The HBV genome is approximately 3.2 kb in length, with four overlapping open reading frames encoding the polymerase, core, X protein, and surface antigen. The surface antigen is a multifunctional glycoprotein composed of three subunits: large (LHB), middle (MHB), and small (SHB) subunits. The three subunits have the same N-glycosylation pattern, which regulates their folding, degradation and function (Dobrica et al., 2020). The presence of SERINC5 results in an decrease in the glycosylation status of the LHB, MHB, and SHB proteins and causes a slight reduction in the levels of HBs proteins, consequently leading to a decrease in HBV secretion (Liu et al., 2020) (**Figure 2**). The co-localization of SERINC5 with LHB proteins within the Golgi apparatus is crucial for decreasing glycosylated LHB (Liu et al., 2020).

SERINC5 is a transmembrane protein characterized by ten putative transmembrane domains, with the tenth domain deemed non-essential for its inhibitory function against hepatitis B virus (HBV) but critical for the protein’s stable expression. Research indicates that fragments 1–253 and 145–253 exhibit instability, while either fragment of 1–145, 145–311 and 145–253 loses the capacity to inhibit HBV, and facilitates the production of glycosylated large and middle hepatitis B antigens (LHB and MHB). Additionally, two conserved N-glycosylation sites, N133 and N294, do not play a role in HBV restriction. It is noteworthy that the fourth to sixth transmembrane domains of SERINC5 are essential for the reduction of glycosylation (Liu et al., 2020). Nevertheless, there exists a paucity of information concerning the effects of HBV on SERINC5 expression levels both *in vitro* and *in vivo*.

### 3.4 CSFV

Classical swine fever is a contagious viral disease affecting domestic and wild pigs. The pathogen responsible for classical swine fever is CSFV. CSFV is an enveloped virus with a positive-sense, single-stranded RNA genome belonging to the *Pestivirus* genus within the *Flaviviridae* family. SERINC5 does not directly target the viral life cycle to inhibit CSFV replication, while SERINC5 inhibits CSFV by interacting with the RNA sensor protein MDA5 and enhancing the MDA5-dependent IFN-I response (Li et al., 2020). In addition, SERINC5 binds with IFN-induced transmembrane proteins 1/2/3 to inhibit viral replication and regulate the NF-κB signaling pathway (Li et al., 2022b) (**Figure 2**). In cultured cells, CSFV infection has been shown to decrease the levels of endogenous SERINC5 in a time- and dose-dependent manner, as evidenced by western blot analysis using a specific SERINC5 antibody and real-time PCR to assess mRNA levels (Li et al., 2020). This experimental approach, utilizing a specific SERINC5 antibody, facilitates the detection and quantification of SERINC5 levels post-infection, thereby elucidating the role of SERINC5 in viral infections. Notably, most prior studies have focused on the function of SERINC5 through exogenous expression due to the lack of a specific SERINC5 antibody (Passos et al., 2019). Although CSFV infection reduces SERINC5 production in cultured cells *in vitro* and in tissues from CSFV-infected pigs *in vivo*, no studies have examined which viral factor affects the level of SERINC5 (Li et al., 2020).

### 3.5 Sendai virus

Sendai virus is an enveloped virus with a negative-strand RNA genome belonging to the *Respirovirus* genus of the *Paramyxovirinae* subfamily (Russell and Hurwitz, 2016). There are no studies on the direct inhibition of the Sendai life cycle by SERINC5. SERINC5 restricts Sendai replication by regulating the immune response. SERINC5 interacts with MAVS and TRAF6 to form the complex of SERINC5/MAVS/TRAF6, and the complex leads to MAVS aggregation and K63-linked polyubiquitylation of TRAF6, which activates NF-κB signaling and then produces IFNs, which exert antiviral effects (Zeng et al., 2021) (**Figure 2**). However, there are no reports about the regulation of SERINC5 by Sendai virus.

## 4 Future directions and conclusions

Pathogens, the microorganisms responsible for diseases, are classically categorized into several groups including bacteria, viruses, fungi, parasites, and prions. Among these entities, SERINC5 has emerged as a significant player in antiviral defense, demonstrating its efficacy against various viruses, including HIV, EIAV, MLV, IAV, SARS-CoV-2, HBV, CSFV, and the Sendai virus (Rosa et al., 2015; Usami et al., 2015; Ahi et al., 2016; Chande et al., 2016; Li et al., 2020; Liu et al., 2020; Zeng et al., 2021; Lai et al., 2022; Timilsina et al., 2022; Zhao et al., 2022) (**Table 1**). The remarkable breadth of SERINC5’s antiviral activity positions it as a pivotal subject of interest in virology. Despite the promising insights into SERINC5’s role in viral infections, there exists a conspicuous lack of research examining its potential impact on bacterial, parasitic, and fungal infections. This notable gap in the literature presents an invaluable opportunity for further investigation, which can enrich our understanding of host-pathogen dynamics and highlight the multifaceted roles SERINC proteins.

**Table 1:** The mechanism by which SERINC5 inhibits virus replication.

Protein	Genus	Species	Gene	Enveloped virus	Function and reference
SERINC5	Lentivirus	HIV	RNA	Enveloped	Blocking virus−cell fusion (Rosa et al., 2015; Usami et al., 2015; Sood et al., 2017; Zhang et al., 2019; Chen et al., 2020; Featherstone and Aiken, 2020; Ward et al., 2020; Leonhardt et al., 2023). Inhibiting genome replication (Chiu et al., 2001; Ramdas and Chande, 2023; Shi et al., 2023) Inhibiting viral release (Li et al. 2019b) Spuring inflammatory regulation (Beitari et al., 2017; Pierini et al., 2021; Zeng et al., 2021)
SERINC5	Influenza virus A	IAV	RNA	Enveloped	Blocking virus-cell fusion (Lai et al., 2022; Zhao et al., 2022)
SERINC5	Beta coronavirus	SARS-CoV-2	RNA	Enveloped	Blocking virus-cell fusion (Timilsina et al., 2022)
SERINC5	Orthohepadnavirus	HBV	DNA	Enveloped	Decreasing HBV secretion (Liu et al., 2020)
SERINC5	Pestivirus	CSFV	RNA	Enveloped	IFN response (Li et al., 2020) Upregulating NF-κB signal (Li et al., 2022b) Inhibiting autophagy (Li et al., 2022b)
SERINC5	Respirovirus	Sendai virus	RNA	Enveloped	Upregulating IFN and NF-κB signal (Zeng et al., 2021)

Viruses can be classified according to the different characteristics, such as their genetic material, infected host, and envelope coating. SERINC5 has antiviral activity against viruses with different genetic materials (Rosa et al., 2015; Liu et al., 2020) and different infected hosts (Rosa et al., 2015; Ahi et al., 2016; Chande et al., 2016), whereas all viruses restricted by SERINC5 have envelope coatings (Rosa et al., 2015; Ahi et al., 2016; Chande et al., 2016; Liu et al., 2020), highlighting that envelope coatings might be the main target for SERINC5. However, the effect of SERINC5 on nonenveloped viruses should be investigated in the future, to confirm further the importance of enveloped coatings in the course of SERINC5-restricted viruses.

Moreover, SERINC5 is embedded into the virions of HIV (Rosa et al., 2015; Usami et al., 2015) and SARS-CoV-2 (Timilsina et al., 2022) to inhibit viral entry (**Table 1**), whereas SERINC5 is located in the plasma membrane of infected cells to inhibit IAV entry (Lai et al., 2022; Zhao et al., 2022). Therefore, SERINC5 either in virion or in the plasma membrane restricts viral entry, and there is likely some interaction of SERINC5 in virions with SERINC5 in the plasma membrane. Moreover, SERINC5 either in the virion or in the plasma membrane mainly acts on viral envelope proteins, such as HIV Env (Rosa et al., 2015; Usami et al., 2015), SARS-CoV-2 S (Timilsina et al., 2022), and IAV HA (Lai et al., 2022; Zhao et al., 2022), to inhibit viral entry. The common characteristics are that HIV-1 Env, SARS-CoV-2 S and IAV HA are cleaved into an extracellular subunit (gp120, S1, HA1) and a transmembrane subunit (gp41, S2, HA2), respectively, before being released from virus producing cells (Jackson et al., 2022). Additionally, HIV Env (Zhang et al., 2019; Chen et al., 2020), IAV HA (Weis et al., 1988; Gamblin et al., 2004), and SARS-CoV-2 S (Jackson et al., 2022) exist as a trimer [(gp120/gp41)3, (S1/S2)3, (HA1/HA2)3] on the viral surface and undergo conformational changes during viral entry. However, it is not clear about the similarities between HIV Env, SARS-CoV-2 S and IAV HA, which may provide clues for a deeper understanding of how SERINC5 targets viral envelope proteins, and that should be explored further in the future.

To date, SERINC5 has been shown to impact the fusion of the virion−plasma membrane, primarily in HIV (Rosa et al., 2015; Usami et al., 2015), SARS−CoV−2 (Timilsina et al., 2022) and IAV (Lai et al., 2022; Zhao et al., 2022), but for HBV, CSFV and Sendai virus, virion−plasma membrane fusion has not been detected. In the next step, more studies should focus on whether SERINC5 inhibits the fusion of the HBV, CSFV and Sendai viruses with the membrane, which will help us reveal the general pattern of virion-membrane fusion.

Upon viral infection, pathogen recognition receptors (PRRS) recognize viral DNA or RNA and trigger the production of IFN-I (INF–α and IFN-β), which is the body’s first line of defense against pathogen infection and has the key role in driving antiviral innate and adaptive immunity to clear pathogens. IFN-I, in turn, increases PRR expression, which is a positive feedback loop to fight against pathogen invasion (Schlee and Hartmann, 2016; Zeng et al., 2021). In recent years, SERINC5 has been shown to increase the production of IFN-I (INF–α and IFN-β) through binding with IFN-induced transmembrane proteins 1/2/3 after CSFV infection (Li et al., 2022b), SERINC5 also is proved to increase the production of IFN-I (INF–α) after HIV infection (Passos et al., 2019), and SERINC5 also increases the production of IFN-I (INF–α and IFN-β) through interacting with the MAVS and TRAF6 proteins after Sendai virus infection (Zeng et al., 2021). Knock out of SERINC5 reduces the mRNA amount of IFN-α, IFN-β, IL-6, and TNF-α in cells infected with Sendai virus or treated with poly(I:C); and knock out of SERINC5 also decreases the amount of mRNA encoding IFN-β, TNF-α, IL-1β, and IL-8 upon lipopolysaccharide stimulation. But without viral infection or immune response stimulation, SERINC5 has no significant effect on the production of inflammatory factors (Zeng et al., 2021). Therefore, under the environment of infection and immune-response trigger treatment, SERINC5 promotes the production of IFN-I and downstream multiple inflammatory factors (Zeng et al., 2021). SERINC5 has stronger anti-viral ability in wild type cells than in IFN I–deficient cells (Zeng et al., 2021). Therefore, SERINC5 triggers immune response in addition to its direct antiviral ability. In turn, although IFN–α treatment can increase the amounts of SERINC5 at the surface of T cells (Passos et al., 2019), it does not increase SERINC5 mRNA levels or protein levels, which leads to that SERINC5 is generally considered a nonclassical PRR (Usami et al., 2015; Xu et al., 2022; Wang et al., 2023). However, it is noted that INF–α does not increase the SERINC5 mRNA level, which occurs in cells without viral infection (Usami et al., 2015; Xu et al., 2022; Wang et al., 2023). It is reported that SERINC5 interacts with MAVS and enhances the formation of MAVS polymers at mitochondria after virus infection, which recruits TRAF6, are essential for NF-κB signaling (NF-κB is the transcription for IFN in response to viral infections and immune responses). Furthermore, the relative abundance of SERINC5 is increased in the presence of MAVS and TRAF6, while with increasing amounts of SERINC5 in this mitochondria, the relative amount of MAVS and TRAF6 at mitochondria appear to increase accordingly (Zeng et al., 2021). Therefore, in the complex of SERINC5, MAVS and TRAF6, SERINC5 forms positive feedback loop with MAVS and TRAF6. However, it must be emphasized that the effect of SERINC5 on MAVS oligomerization is dependent on Sendai infection or ligand stimulation, because MAVS oligomerization itself does not always lead to the formation of a functional complex (Zeng et al., 2021). Therefore, the positive loop of IFN-I, MAVS and TRAF6 is dependent on viral infection, and the effect of INF-I on SERINC5 mRNA after various viruses infection should be investigated more in the future, possibly, upon viral infection INF-I can increase the level of SERINC5, and possibly there is a positive feedback loop between SERINC5 and IFN-I.

To antagonize SERINC5, different viruses utilize different viral proteins, such as Nef (Aiken, 2015; Rosa et al., 2015; Usami et al., 2015; Shi et al., 2018) and Env (Rosa et al., 2015; Usami et al., 2015; Beitari et al., 2017; Zhang et al., 2019; Timilsina et al., 2020) of HIV, S2 of EIAV (Chande et al., 2016), glycosylated Gag of MLV (Li et al., 2019b), ORF7a of SARS-CoV-2 (Timilsina et al., 2022), or the viral core of HIV (Diehl et al., 2021) and svRNA of SARS-CoV-2 (Meseguer et al., 2023), to decrease the level or function of SERINC5 (**Table 2**). Although there is no similar sequence among Nef, glycosylated Gag and S2, they share two similar loci, one of which is the site of myristoylation located at the N-terminus; the other is the dileucine motif, and the two loci are important for degrading SERINC5. SERINC5 is degraded through endolysosome pathway, inhibiting the lysosomal and endocytic pathways increases SERINC5 expression and function (Aiken, 2015; Rosa et al., 2015; Usami et al., 2015; Shi et al., 2018), therefore inhibiting the lysosomal and endocytic pathways should be considered before targeting SERINC5 for antivirus drug development. ORF7a of SARS-CoV-2 counteracts SERINC5 because it prevents the incorporation of SERINC5 into SARS-CoV-2 virions in producer cells, and in virions ORF7a, S and SERINC5 form a complex, the complex restricts the antiviral effect of SERINC5 during virus-cell membrane fusion. Although ORF7a inhibits SERINC5 function, ORF7a does not affect the expression of SERINC5 (Timilsina et al., 2022). SARS-CoV-2 svRNA mainly decreases SERINC5 mRNA level. Therefore, viral factors from different viruses inhibit SERINC5 expression and function from various perspectives. For IAV, HBV, CSFV and Sendai virus, there are no reports about viral factors antagonizing SERINC5; in the future, more studies should focus on how these viruses using own viral factor(s) to resist SERINC5, which may reveal more unexpected mechanisms.

**Table 2:** Viral factor(s) antagonizing SERINC5.

Virus	Virus factor	Target factor	Function
HIV	Nef	SERINC5	Blocking the incorporation of SERINC5 in virion (Aiken, 2015; Rosa et al., 2015; Usami et al., 2015; Shi et al., 2018) Degrading SERINC5 (Aiken, 2015; Rosa et al., 2015; Usami et al., 2015; Shi et al., 2018; Staudt and Smithgall, 2020)
	Env	SERINC5	Overcoming SERINC5 restriction (Rosa et al., 2015; Usami et al., 2015; Beitari et al., 2017; Zhang et al., 2019; Timilsina et al., 2020)
	Viral core	SERINC5	Antagonizing SERINC5 function (Diehl et al., 2021)
Murine leukemia virus	Glycosylated Gag	SERINC5	Blocking the incorporation of SERINC5 in virion (Chande et al., 2016) Degrading SERINC5 (Chande et al., 2016)
Equine infectious anemia virus	S2	SERINC5	Blocking the incorporation of SERINC5 in virion (Li et al., 2019b) Degrading SERINC5 (Li et al., 2019b)
IAV	HA	SERINC5	HA glycosylation affecting SERINC5’s anti-IAV ability (Weis et al., 1988)
SARS-CoV-2	ORF7a	SERINC5	Blocking the incorporation of SERINC5 in virion (Timilsina et al., 2022)
	SvRNAs	SERINC5	Inhibiting SERINC5 expression (Meseguer et al., 2023)
HBV	No data	SERINC5	No data
CSFV	Viral infection	SERINC5	Decreasing SERINC5 level (Li et al., 2020)
Sendai virus	No data	SERINC5	No data

Host factors support the ability of SERINC5 to restrict viruses, but viruses usually hijack them for replication. Host factors, such as cell differentiation (Zutz et al., 2020) and Cullin3-KLH20 (Li et al., 2022a) regulate the stability and the function of SERINC5 (**Table 3**). The presence of CD4 helps SERINC5 dissociate Env trimer to improve viral sensitivity to SERINC5, further blocking viral entry (Zhang et al., 2019), and it is interesting that both CD4 and SERINC5 are degraded by HIV Nef (Piguet et al., 1999; Aiken, 2015; Rosa et al., 2015; Usami et al., 2015; Shi et al., 2018). Therefore, the relationship of SERINC5 with CD4 should be investigated further which provides the clues for HIV treatment and drug development targeting both CD4 and SERINC5. HA low glycosylation at important domain increases the sensitivity of IAV to SERINC5 restriction (Zhao et al., 2022), whereas SERINC5 decreases the glycosylation level of HBV proteins (Liu et al., 2020); therefore, SERINC5 decreases the glycosylation of viral proteins to increase viral sensitivity to SERINC5 restriction, which might apply to various viruses. The effect of SERINC5 on viral protein glycosylation may be another main antiviral mechanism because viral glycoproteins are important for viral protein expression, fusion, binding with cell receptors, virulence and so on (Harrison, 2008; Bowden et al., 2011; Liu and Yang, 2021; Feng et al., 2022).

**Table 3:** The regulation between host factors and SERINC5 after virus infection.

Virus	Host factor	Function
HIV	EDTEE of SERINC5	Improving SERINC5 stability (Stoneham et al., 2020)
	N294 of SERINC5	Improving SERINC5 steady expression (Sharma et al., 2018)
	Aromatic side chain at 412 of SERINC5	Upregulating anti-HIV ability of SERINC5 (Tan et al., 2021)
	F397 of SERINC5	Keeping anti-HIV ability of SERINC5 (Stoneham et al., 2020)
	IFN-I	Enhancing the membrane surface level of SERINC5 (Passos et al., 2019)
	Cell differentiation of myeloid lineage	Upregulating SERINC5 level (Zutz et al., 2020)
	CD4 expression	Helping SERINC5 restricting Env (Zhang et al., 2019)
	CDK13 binding with SERINC5, and CyclinK/CDK13 phosphorylating SERINC5	Helping Nef degrade SERINC5 (Chai et al., 2021)
	Cullin3-KLH20 regulating SERINC5 ubiquitination	Promoting SERINC5 on plasma membrane or downregulating SERINC5 (Li et al., 2022a)
IAV	K130 of SERINC5	Keeping SERINC5 localization in the plasma membrane (Lai et al., 2022)
CSFV	Interacting with interferon-induced transmembrane proteins 1/2/3	SERINC5 enhancing MDA5-mediated IFN-I response (Li et al., 2020)
HBV	N133 and N294 are two conserved N-glycosylation sites of SERINC5	Keeping SERINC5’s glycosylation (Liu et al., 2020)
	1–145, 145–311 and 145–253 of SERINC5	Either of them has no ability of anti-HBV (Liu et al., 2020)
Sendai virus	Interacting with mitochondrial antiviral signaling protein(MAV)	Helping SERINC5 improving IFN and NF-κB signal (Zeng et al., 2021)
	Forming the complex with tumor necrosis factor receptor-associated factor6 (TRAF6)	

In homeostasis, the expression levels of SERINC proteins are finely regulated through transcriptional and post-transcriptional mechanisms. Unfortunately, no studies have investigated the transcription factors involved in SERINC expression. Additionally, SERINC5 steady-state levels increase after treatment with the proteasome inhibitor MG132 or the lysosome inhibitor NH4Cl in the absence of viruses (Zhang et al., 2017; Ahmad et al., 2019). S2 of EIAV again reduces SERINC5 expression at steady-state levels, and this effect is partially blocked by NH4Cl but not by MG132 (Ahmad et al., 2019). The findings indicate that in the absence of viral presence, SERINC5 undergoes degradation primarily via the proteasome and lysosomal pathways. Conversely, in the presence of viruses, the degradation of SERINC5 is predominantly localized to the lysosome. This distinction underscores the influence of viral factors on the regulatory mechanisms governing SERINC5 stability and degradation. Meanwhile the regulation of SERINC5 expression is a multifaceted process influenced by various factors, as evidenced by contrasting findings in different studies. Timilsina et al. (Timilsina et al., 2022) observed no significant effect of SARS-CoV-2 infection on SERINC5 transcription levels in Calu-3 cells, while Meseguer et al. (Timilsina et al., 2022) reported a reduction in SERINC5 mRNA levels in COVID-19 patients, with notable decreases at 4 hours post-infection in Vero E6 and HEK293T-hACE2 cells, as well as a decline in protein levels at 16 hours post-infection. Additionally, CSFV infection has been shown to downregulate SERINC5 expression (Li et al., 2020), and a similar downregulation was noted in HIV-1-infected patients (Hernández-López et al., 2021). These findings underscore the complexity of SERINC5 regulation, highlighting the necessity for further research into the molecular pathways that govern its expression to better understand its roles in disease pathology. A visual representation of these regulatory mechanisms may enhance comprehension and facilitate the dissemination of this critical information.

In summary, accumulating evidence suggests that SERINC5, which was initially identified as an antiretroviral restriction factor, has broad antiviral activity against various viruses from different virus families. We suggest that SERINC5 should be expressed via genetic engineering and delivered into cells to inhibit retroviruses and nonretroviruses. Ultimately, conducting more in-depth investigations on the interplay between viruses and SERINC5 will lead to a more precise understanding of the antiviral function of SERINC5 and viral pathogenicity.
